# Risk Stratification to Optimize Colorectal Cancer Screening: Development and Validation of a Decision‐Tree Model for Colonoscopy Prioritization

**DOI:** 10.1111/den.70224

**Published:** 2026-07-15

**Authors:** María Capilla‐Lozano, Carmen Quiñones‐Torrelo, Lucas Sebastián‐Peris, José Miguel Carot‐Sierra, Concepción Gómez‐Medina, Inmaculada Lorca, Clara Alfaro‐Cervelló, Pilar Aguilar, Susana Castán‐Cameo, Silvia Gimeno‐Martos, Marta Ponce‐Romero, Isabel Pascual‐Moreno, Arturo Carratalá‐Calvo, Andrés Peña, David Martí‐Aguado

**Affiliations:** ^1^ Digestive Disease Department Clinic University Hospital of Valencia, INCLIVA Health Research Institute Valencia Spain; ^2^ Digestive Disease Department Hospital Universitario de La Plana Vila‐real Spain; ^3^ Clinical Biochemistry Department Clinic University Hospital of Valencia, INCLIVA Health Research Institute Valencia Spain; ^4^ Department of Applied Statistics Operations Research and Quality, Universitat Politècnica de València Valencia Spain; ^5^ Pathology Department Clinic University Hospital of Valencia, INCLIVA Health Research Institute Valencia Spain; ^6^ Faculty of Medicine and Dentistry, University of Valencia Valencia Spain; ^7^ Valencia Department of Universal Healthcare and Public Health Valencia Spain

**Keywords:** colorectal neoplasms, early detection of cancer, fecal occult blood test, mass screening, risk assessment

## Abstract

**Background and Aims:**

Fecal immunochemical test‐based colorectal cancer screening is effective; however, colonoscopy demand exceeds endoscopy capacity. Delays in colonoscopy after a positive test reduce screening effectiveness. We developed and validated a decision‐tree model to stratify colorectal cancer risk among individuals with a positive test to prioritize colonoscopy for those at highest risk.

**Methods:**

Prospective study including participants with a positive fecal immunochemical test referred for colonoscopy within a Spanish regional population‐based screening program (2021–2024). Individuals were randomly assigned to derivation (65%) and validation (35%) cohorts using a stratified random split. Fecal hemoglobin concentration, clinical and laboratory data were collected to develop the decision‐tree model. The primary outcomes were colorectal cancer and advanced‐stage colorectal cancer.

**Results:**

A total of 1773 participants were included. Colorectal cancer was detected in 97 participants (5.5%), including 25 cases (1.5%) with advanced‐stage disease. The model stratified participants into low‐, intermediate‐, high‐, and very high‐risk groups. A progressive increase in the prevalence of colorectal cancer and advanced‐stage disease was observed across risk groups in both cohorts (*p* < 0.001). The very high‐risk group (fecal hemoglobin > 100 μg/g and > 65 years) included 6.7% of participants, with colorectal cancer and advanced‐stage prevalence of 21.8% and 7.6%, respectively. Prioritizing colonoscopy for this group yielded a number needed to scope of 4.6 for colorectal cancer and 13.2 for advanced‐stage disease, both significantly lower than the current one‐size‐fits‐all strategy.

**Conclusions:**

A decision‐tree model was developed to stratify the risk of colorectal cancer and optimize endoscopic resources in screening programs.

## Graphical abstract

In fecal immunochemical test (FIT)‐based colorectal cancer (CRC) screening, colonoscopy demand frequently exceeds endoscopic capacity, and delays after positive test are associated with CRC stage progression and poorer clinical outcomes. A decision‐tree algorithm integrating fecal hemoglobin concentration, age, alkaline phosphatase and carcinoembryonic antigen was developed, enabling identification of very high‐risk and low‐risk CRC subgroups among FIT‐positive individuals. Risk‐based stratification of FIT‐positive individuals may support prioritized colonoscopy for patients at highest risk, improving efficiency when endoscopic capacity is limited.

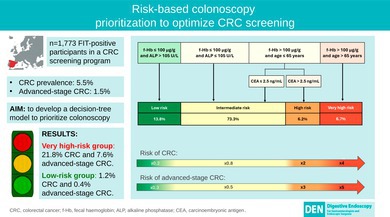

**Abbreviations**
ALP: alkaline phosphatase
ALT: alanine aminotransferase
AST: aspartate aminotransferase
BMI: body mass index
CART: classification and regression tree
CEA: carcinoembryonic antigen
CI: confidence interval
CRC: colorectal cancer
f‐Hb: fecal hemoglobin
FIT: fecal immunochemical test
FN: false negative
FP: false positive
GGT: gamma‐glutamyl transferase
HDL‐C: high‐density lipoprotein cholesterol
INR: international normalized ratio
IQR: interquartile range
LDL‐C: low‐density lipoprotein cholesterol
LR: likelihood ratio
NNS: number needed to scope
NPV: negative predictive value
OR: odds ratio
PPV: positive predictive value
sTfR: soluble transferrin receptor
TRIPOD: transparent reporting of a multivariable prediction model for individual prognosis or diagnosis

## 1 Introduction

Colorectal cancer (CRC) is a major global health problem, ranking as the third most frequent malignancy worldwide and the second leading cause of cancer‐related mortality [1, 2]. Prognosis depends on the tumor stage at diagnosis [3]. To improve early detection and outcomes, population‐based screening programs have been widely implemented, with fecal immunochemical test (FIT) being the preferred screening test across Europe [4].

The effectiveness of FIT‐based screening depends on timely undertaking colonoscopy in FIT‐positive participants [5, 6]. Delays in post‐FIT colonoscopy are significantly associated with CRC stage progression and poorer clinical outcomes [5, 6]. Therefore, European guidelines recommend completing colonoscopy within 1 month of a positive FIT as a key quality indicator [7]. Within this time frame, the COLONPREV trial demonstrated that a FIT‐based strategy is non‐inferior to colonoscopy in reducing CRC‐related mortality [8].

Ensuring timely colonoscopy has become an increasing organizational challenge due to multiple factors. These include limited endoscopic capacity, increasing demand from post‐polypectomy surveillance, higher screening adherence [9], and the impact of the COVID‐19 pandemic, which has significantly increased the waiting lists for endoscopic procedures and delayed CRC diagnosis [10, 11].

To address these challenges, scientific societies advocate for risk‐based CRC stratification, enabling the prioritization of colonoscopies in FIT‐positive individuals [12]. Quantitative fecal hemoglobin (f‐Hb) concentration on FIT has emerged as a useful stratification tool, as higher f‐Hb correlates with advanced colorectal neoplasia [13, 14, 15]. A recent study from Italy proposed a model‐based algorithm incorporating f‐Hb and additional variables to estimate the probability of CRC [16]. While innovative, this predictive model has some limitations that may affect its implementation in screening programs. First, although sex is a well‐established predictor of CRC risk, its use as a prioritization criterion warrants careful consideration as it may compromise equitable allocation of limited endoscopic resources. Second, 23% of participants were classified as high risk, a proportion too large to be realistically prioritized. Third, important variables such as CRC stage and relevant clinical or analytical data were not fully characterized.

Risk‐based cancer screening seeks to replace one‐size‐fits‐all recommendation to optimize healthcare resources while preserving clinical benefit [12, 17]. Applied to CRC screening, this paradigm offers the potential to prioritize diagnostic colonoscopies for those individuals most likely to benefit, without compromising equity. The current study aims to optimize CRC screening program by developing and validating a decision‐tree algorithm to identify individuals at very high risk and at low risk of CRC after a positive FIT, enabling a more rational and equitable allocation of colonoscopy resources.

## 2 Methods

### 2.1 Design and Population

A prospective, single‐center study was conducted between April 2021 and March 2024. The study population comprised participants in the CRC Screening Program of the Valencia Clinic‐Malvarrosa Department of Health (Valencia, Spain). All individuals aged 50–70 years undergoing colonoscopy after a positive FIT were invited to participate. Non‐eligible patients included those with a history of CRC surgery or diagnosis of Inflammatory Bowel Disease. Inadequate or incomplete colonoscopies were excluded because these examinations are not reliable for evaluating endoscopic findings. Although repeat colonoscopy was performed, it generally occurred 1 year after the screening FIT, and findings in such cases may no longer be related to the initial f‐Hb level [16]. Oral anticoagulant users were also excluded because they represent a distinct subpopulation with higher FIT‐positivity and lower positive predictive value (PPV) for CRC after a positive FIT, as demonstrated in other national screening programs [18, 19]. The study protocol was approved by the Institutional Ethical Committee (Registry#:2021/373) and complied with the Declaration of Helsinki. Written informed consent was obtained from each participant.

### 2.2 Diagnostic Tests and Definitions

FIT analysis was performed using a single stool sample with OC‐Sensor Pledia (Eiken Chemical, Tokyo, Japan), in accordance with the standard strategy of the Spanish population‐based screening program [8, 20]. A result was considered positive if f‐Hb ≥ 20 μg/g and the upper quantification limit was 200 μg/g [12, 20]. Prior to colonoscopy, participants completed a medical questionnaire and underwent blood testing (see Supporting Information). All colonoscopies were performed by experienced endoscopists (> 100 colonoscopies/year) and fulfilled the technical quality standards according to guidelines [7, 20, 21]. All detected lesions were biopsied or removed and evaluated histologically. Some participants underwent more than one colonoscopy due to complex polyps. Lesions were classified according to the Vienna Classification [22]. CRC was classified as early (I–II) or advanced (III–IV) stage, according to TNM classification.

### 2.3 Endpoints

The primary endpoint was the development and internal validation of a decision‐tree algorithm to stratify CRC risk among FIT‐positive individuals within a population‐based screening program. The main outcomes evaluated were the diagnosis of CRC and advanced‐stage CRC at colonoscopy, with histological confirmation. The intended implication of the algorithm was to prioritize colonoscopy in individuals classified as very high risk, while identifying individuals at comparatively lower risk in whom timing to colonoscopy might be individualized.

### 2.4 Statistical Analysis

Analyses were performed using SPSS Statistics V25 and Python 3.12. Comparisons between groups are detailed in Supporting Information. All statistical tests were two‐sided, considering *p* < 0.05 as statistically significant.

**FIGURE 1:**
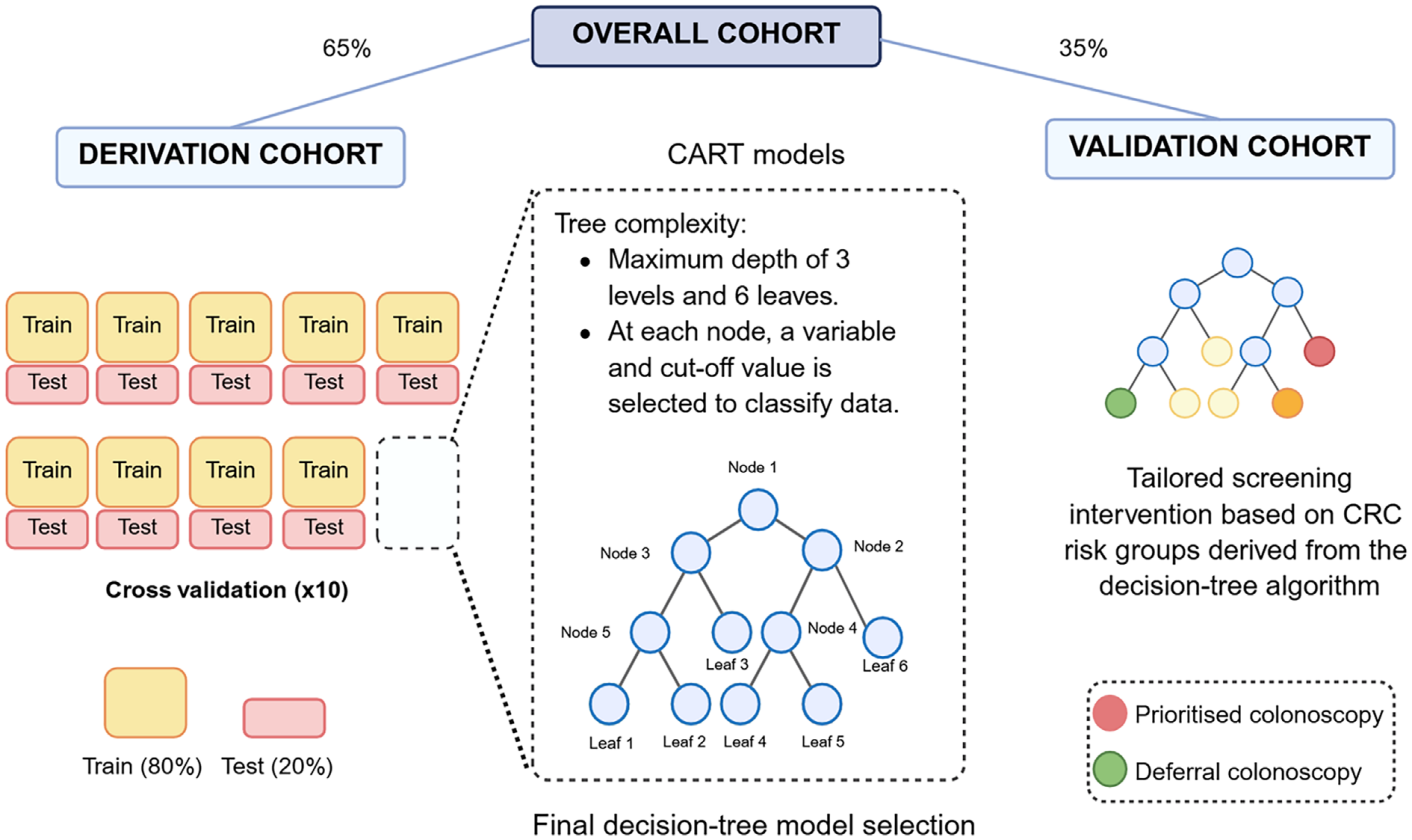
Study design and development of the decision‐tree model. Study design showing cohort division and development of the classification and regression tree (CART) model using cross‐validation. CART, classification and regression tree; CRC, colorectal cancer.

The sample was divided into derivation and validation cohorts using stratified randomization. Participants were randomly assigned in a 65:35 ratio within strata defined by age group, thereby preserving the distribution of this variable across both cohorts (Figure 1). A fixed random seed ensured reproducibility. Classification and regression tree (CART) models were constructed with binary splits using iterative data partitioning to identify variable pathways and cutoff values associated with CRC. Model training optimized log‐loss function (negative log‐likelihood) to improve probability calibration and enable the application of decision thresholds other than conventional 0.5 in downstream risk‐stratification. To enhance model interpretability and minimize overfitting, the tree complexity was constrained a priori to a maximum depth of three levels and six leaves or terminal nodes. Model optimization was performed using a 10‐fold cross‐validation within the derivation cohort. Each fold used 80% of data for training and 20% for testing, generating 10 candidate decision‐trees. For each terminal node, the algorithm computed a model‐estimated probability of CRC (weighted probability within that node). The proportions of the risk groups were prespecified based on precedents policy statements for risk‐stratified screening in other cancer settings [23]. Specifically, 5%–10% of participants were classified as very high risk, while 15%–20% were classified as low risk. The final decision‐tree model selection was based on performance in the validation cohort, prioritizing maximal sensitivity for CRC detection within the high‐risk group, consistent with the intended use of the algorithm for risk‐based screening prioritization [24].

Then, the decision‐tree algorithm was applied in the derivation, validation, and overall study cohorts to calculate the observed prevalence of CRC and advanced‐stage CRC across risk‐groups. Diagnostic performance metrics included: sensitivity, specificity, false positive (FP) and false negative (FN) rates, PPV, negative predictive value (NPV), percent correctly classified, odds ratio (OR), likelihood ratios (LR+, LR‐), and the number needed to scope (NNS), defined as the number of colonoscopies required to detect one case of CRC [15]. For reference, previously reported NNS values among FIT‐positive screening participants are approximately 18 for CRC and 110 for advanced‐stage CRC [25]. Participants with missing clinical or analytical predictors required for the decision tree algorithm (*n* = 122) were imputed using a k‐nearest neighbors' approach (*k* = 7). There was no missing data for outcomes.

## 3 Results

### 3.1 Population Characteristics

**FIGURE 2:**
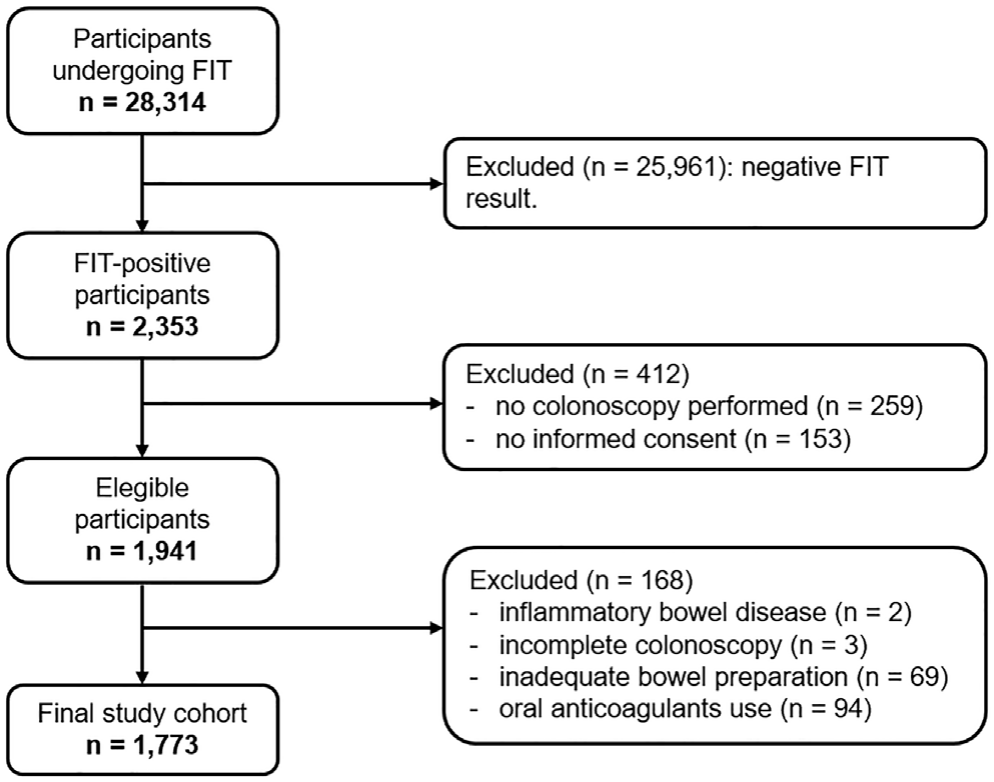
Flow diagram of participant selection. Flowchart of inclusion and exclusion of participants undergoing fecal immunochemical test (FIT) screening. FIT, fecal immunochemical test.

During the study period, 28,314 participants underwent FIT, of whom 2353 (8.3%) tested positive. All FIT‐positive individuals were referred for colonoscopy, with 1941 attending the procedure and providing informed consent for inclusion. After applying exclusion criteria, the final study population comprised 1773 participants (Figure 2). Median interval between FIT‐positive and colonoscopy was 3 [2−4] months. Colonoscopy was performed within 1 month in 41 participants (2.3%), 1–3 months in *n* = 930 (52.5%), 3–6 months in *n* = 706 (39.8%), and > 6 months in *n* = 95 (5.4%).

The derivation (*n* = 1167) and validation (*n* = 606) cohorts were comparable regarding clinical characteristics and endoscopic findings (Table 1). Overall, CRC was diagnosed in 97 patients (5.5%), of whom 25 (25.8%) had advanced‐stage disease. Compared to colonoscopy performed within < 1 month, the rate of CRC and advanced‐stage CRC numerically increased with colonoscopy delay (Table S1). Participant characteristics according to CRC status are presented in Table S2. The prevalence of CRC and advanced‐stage CRC significantly increased across f‐Hb concentration (Table S3).

**TABLE 1:** Baseline characteristics and endoscopic findings of the overall, derivation, and validation cohorts.

Variable	Overall cohort (*n* = 1773)	Derivation cohort (*n* = 1167)	Validation cohort (*n* = 606)
Female sex	856 (48.3%)	586 (50.2%)	270 (44.6%)
Age (years)	61 (57–66)	61 (56–65)	63 (58–67)
Tobacco			
Nonsmoker	874 (49.3%)	590 (50.6%)	284 (46.9%)
Ex‐smoker	401 (22.6%)	253 (21.7%)	148 (24.4%)
Current smoker	498 (28.1%)	324 (27.8%)	174 (28.7%)
BMI (kg/m^2^)	26 (24.1–30.1)	26.7 (24.0–30.1)	27.0 (24.3–30.1)
Comorbidities			
Arterial hypertension	731 (41.2%)	434 (37.2%)	297 (49.0%)
Dyslipidemia	1002 (56.5%)	633 (54.2%)	369 (60.9%)
Diabetes mellitus	275 (15.5%)	154 (13.2%)	121 (20.0%)
Obesity	440 (24.8%)	285 (24.4%)	155 (25.6%)
f‐Hb (μg/g)	47.2 (29.2–111.0)	48.4 (29.6–115.6)	45.6 (28.4–102.2)
Glucose (mg/dL)	91 (82–101)	92 (83–101)	89 (80–99)
Creatinine (mg/dL)	0.80 (0.68–0.93)	0.79 (0.68–0.93)	0.81 (0.69–0.93)
Hemoglobin (g/dL)	14.8 (13.8–15.7)	14.8 (13.8–15.8)	14.8 (13.8–15.7)
Serum iron (μg/dL)	91 (72–113)	89 (72–110)	95 (73–118)
sTfR (mg/L)	1.16 (1.01–1.38)	1.15 (1.00–1.36)	1.20 (1.03–1.41)
Cholesterol (mg/dL)	194 (166–218)	197 (172–220)	183 (153–213)
Triglycerides (mg/dL)	115 (85–158)	117 (87–158)	110 (81–157)
HDL‐C (mg/dL)	54 (46–64)	55 (47–65)	51 (43–61)
LDL‐C (mg/dL)	118 (95–140)	122 (101–143)	108 (85–131)
Bilirubin (mg/dL)	0.64 (0.50–0.88)	0.63 (0.49–0.83)	0.69 (0.50–0.95)
AST (U/L)	24 (21–30)	24 (21–30)	24 (20–30)
ALT (U/L)	21 (17–29)	22 (17–29)	21 (16–29)
GGT (U/L)	26 (19–40)	26 (19–39)	27 (20–42)
ALP (U/L)	78 (66–93)	77 (65–93)	79 (67–94)
Leukocytes (×10^3^/μL)	7.23 (6.11–8.73)	7.23 (6.06–8.69)	7.22 (6.18–8.78)
Platelet count (×10^3^/μL)	250 (210–295)	251 (211–296)	245 (208–293)
INR	1.00 (1.00–1.02)	1.00 (1.00–1.02)	1.00 (1.00–1.02)
CEA (ng/mL)	2.0 (1.6–3.1)	1.9 (1.4–3.1)	2.0 (1.7–3.2)
CRC	97 (5.5%)	71 (6.1%)	26 (4.3%)
Advanced‐stage CRC^a^	25 (1.5%)	16 (1.4%)	9 (1.5%)
Location of CRC			
Proximal colon	28 (1.6%)	22 (1.9%)	6 (1.0%)
Distal colon	33 (1.9%)	24 (2.1%)	9 (1.5%)
Rectum	36 (2.0%)	25 (2.1%)	11 (1.8%)
Adenoma	949 (53.5%)	625 (53.6%)	324 (53.5%)
Villous	265 (14.9%)	175 (15.0%)	90 (14.9%)
Dysplasia	61 (3.4%)	39 (3.3%)	22 (3.6%)
Size ≥ 10 mm	545 (30.7%)	361 (30.9%)	184 (30.4%)
Advanced adenoma^b^	569 (32.1%)	376 (32.2%)	193 (31.8%)
Serrated polyp	362 (20.4%)	230 (19.7%)	132 (21.8%)
Dysplasia	5 (0.3%)	2 (0.2%)	3 (0.5%)
Size ≥ 10 mm	79 (4.5%)	55 (4.7%)	24 (4.0%)
Advanced serrated^c^	81 (4.6%)	55 (4.7%)	26 (4.3%)
Advanced neoplasia^d^	682 (38.5%)	452 (38.7%)	230 (38.0%)
Other lesions			
Diverticular disease	688 (38.8%)	465 (39.8%)	223 (36.8%)
Hemorrhoids	864 (48.7%)	577 (49.4%)	287 (47.4%)

*Note:* Values are presented as median (interquartile range) or as number (%).

Abbreviations: ALP, alkaline phosphatase; ALT, alanine aminotransferase; AST, aspartate aminotransferase; BMI, body mass index; CEA, carcinoembryonic antigen; CRC, colorectal cancer; f‐Hb, fecal hemoglobin; FIT, fecal immunochemical test; GGT, gamma‐glutamyl transferase; HDL‐C, high‐density lipoprotein cholesterol; INR, international normalized ratio; LDL‐C, low‐density lipoprotein cholesterol; sTfR, soluble transferrin receptor.

^a^ Advanced‐stage CRC was defined as stages III–IV.

^b^ Advanced adenoma included lesions ≥ 10 mm, with villous component, or high‐grade dysplasia.

^c^ Advanced serrated polyp included serrated lesions ≥ 10 mm or with dysplasia.

^d^ Advanced neoplasia included advanced adenoma, advanced serrated polyp, or CRC.

### 3.2 Development of the Decision‐Tree Algorithm

The final model incorporated the following variables and cutoff values: f‐Hb 100 μg/g, age 65 years, carcinoembryonic antigen (CEA) 2.5 ng/mL, alkaline phosphatase (ALP) 105 U/L, and body mass index (BMI) 30 kg/m^2^. The model classified participants into six leaves, with estimated CRC weighted probabilities increasing progressively (Figure S1). These weighted probabilities were able to stratify the risk of CRC into four clinically interpretable risk categories: low risk (f‐Hb < 100 μg/g and ALP > 105 U/L), intermediate risk, high risk (f‐Hb > 100 μg/g, ≤ 65 years, and CEA > 2.5 ng/mL), and very high risk (f‐Hb > 100 μg/g and > 65 years).

**FIGURE 3:**
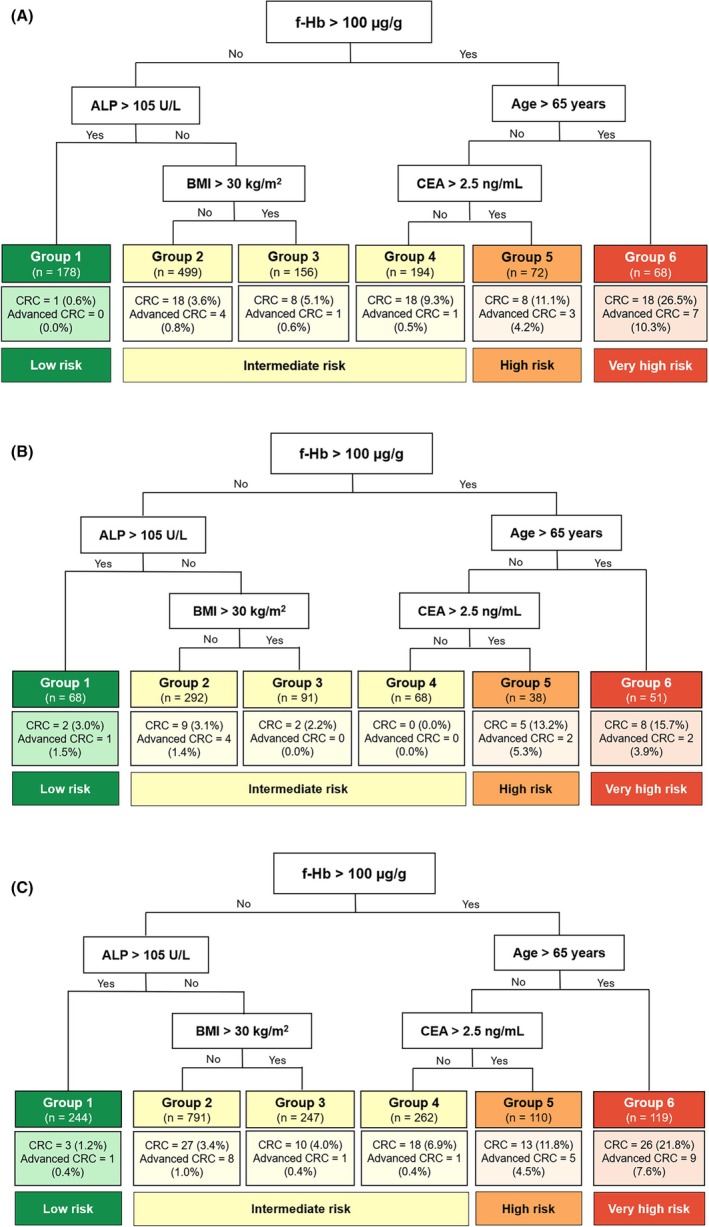
Decision‐tree model for colorectal cancer risk stratification in the derivation (A), validation (B), and overall cohort (C). Decision‐tree model defining six colorectal cancer (CRC) risk groups based on fecal hemoglobin (f‐Hb) and clinical variables. Terminal nodes show the proportion of CRC and advanced‐stage CRC. ALP, alkaline phosphatase; BMI, body mass index; CEA, carcinoembryonic antigen; CRC, colorectal cancer; f‐Hb, fecal hemoglobin.

The corresponding observed prevalence of CRC and advanced‐stage CRC increased progressively across risk groups (*p* < 0.001) (Figure 3A). The very high‐risk group comprised 5.8% of participants (*n* = 68). Compared with all other groups, this subgroup had significantly higher tumor burden, with a CRC prevalence of 26.5% (OR: 7.1, 95% CI: 3.9–13.0) and 10.3% advanced‐stage CRC (OR: 13.9, 95% CI: 5.0–38.6). Prioritizing the very high‐risk patients entails a NNS of 3.8 to diagnose one case of CRC and a NNS of 9.7 to diagnose one case of advanced‐stage CRC. All metrics of the model for CRC diagnosis are detailed in Table 2, and for advanced‐stage CRC in Table S4. The results of prioritizing screening intervention to participants from the high and very high‐risk groups are shown in Tables S5 and S6.

**TABLE 2:** Performance of the decision‐tree model in the very high‐risk group across derivation, validation, and overall cohorts for colorectal cancer.

Diagnostic metrics	Derivation cohort	Validation cohort	Overall cohort
Sensitivity (%)	25.4 (15.8–37.1)	30.8 (14.3–51.8)	26.8 (18.3–36.8)
Specificity (%)	95.4 (94.0–96.6)	92.6 (90.1–94.6)	94.5 (93.3–95.5)
PPV (%)	26.5 (16.5–38.6)	15.7 (7.0–28.6)	21.9 (14.8–30.4)
NPV (%)	95.2 (93.7–96.4)	96.8 (94.9–98.1)	95.7 (94.6–96.6)
Correctly classified (%)	91.2 (89.4–92.7)	89.9 (87.3–92.2)	90.8 (89.3–92.1)
NNS	3.8	6.4	4.6
FP rate (%)	4.6	7.4	5.6
FN rate (%)	74.7	69.2	73.2
LR+	5.6 (3.4–9.0)	4.2 (2.2–7.9)	4.8 (3.3–7.1)
LR—	0.8 (0.7–0.9)	0.8 (0.6–1.0)	0.8 (0.7–0.9)
Odds ratio	7.1 (3.9–13.0)	5.6 (2.3–13.5)	6.2 (3.8–10.2)

*Note:* Values are expressed as percentages (95% confidence interval [CI]) unless otherwise indicated.

Abbreviations: FN, false negative; FP, false positive; LR+, positive likelihood ratio; LR−, negative likelihood ratio; NNS, number needed to scope; NPV, negative predictive value; PPV, positive predictive value.

The low‐risk group comprised 15.3% of the cohort (*n* = 178) and included only one case of CRC, diagnosed at an early stage. Compared with all other groups, this subgroup exhibited a significantly lower tumor burden, with a CRC prevalence of 0.6% (Table 3). Metrics related to considered deferral of screening colonoscopy among individuals classified as low risk are presented in Table S7.

**TABLE 3:** Distribution of colorectal cancer and advanced‐stage colorectal cancer according to risk group derived from the decision‐tree model in the derivation, validation, and overall cohorts.

Outcome	Cohort^a^	Low risk	Intermediate risk	High risk	Very high risk	*p*
CRC	Derivation	1/178 (0.6%)	44/849 (5.2%)	8/72 (11.1%)	18/68 (26.5%)	< 0.001
	Validation	2/66 (3.0%)	11/451 (2.4%)	5/38 (13.2%)	8/51 (15.7%)	< 0.001
	Overall	3/244 (1.2%)	55/1300 (4.2%)	13/110 (11.8%)	26/119 (21.8%)	< 0.001
Advanced‐stage CRC^b^	Derivation	0/178 (0.0%)	6/849 (0.7%)	3/72 (4.2%)	7/68 (10.3%)	< 0.001
	Validation	1/66 (1.5%)	4/451 (0.9%)	2/38 (5.3%)	2/51 (3.9%)	< 0.001
	Overall	1/244 (0.4%)	10/1300 (0.8%)	5/110 (4.5%)	9/119 (7.6%)	< 0.001

*Note:* Values are presented as numerator/denominator and percentage (%).

Abbreviation: CRC, colorectal cancer.

^a^ The derivation, validation, and overall cohorts included 1167, 606, and 1773 participants, respectively.

^b^ Advanced‐stage CRC was defined as stages III–IV.

### 3.3 Validation of the Decision‐Tree Algorithm

Applying the algorithm to an independent validation cohort confirmed the model's trend to stratify participants according to CRC risk (Figure 3B). The very high‐risk group comprised 8.4% of participants (*n* = 51). This subgroup demonstrated a CRC prevalence of 15.7% (OR: 5.6, 95% CI: 2.3–13.5, compared with all other risk groups) and an advanced‐stage CRC prevalence of 3.9% (OR: 3.2, 95% CI: 0.6–15.8). Although CRC prevalence in the very high‐risk group was lower in the validation cohort than in the derivation cohort, the overall risk association across groups was consistent (Table 2 and Table S4). The low‐risk group comprised 10.9% (*n* = 66) of the validation cohort and included two CRC cases, one of which was diagnosed at stage III (see Supporting Information).

The results of the overall cohort (*n* = 1773) are illustrated in Figure 3C. The prevalence of CRC and advanced‐stage CRC were at their lowest in the low‐risk group and progressively increased across groups up to their maximum in the very high‐risk group. In the small subset of participants from the very high‐risk group (6.7% of the study sample), the NNS to diagnose one case of CRC was 4.6 and the NNS to diagnose one case of advanced‐stage CRC was 13.2 (Table 2). The low‐risk group included 13.8% of the cohort (*n* = 244), with CRC and advanced CRC prevalences of 1.2% and 0.4%, corresponding to NNS values of 81.3 and 244, respectively (Table S7). The distribution of CRC and advanced‐stage CRC according to risk groups is presented in Table 3.

## 4 Discussion

The present study addresses a major challenge in FIT‐based screening: the imbalance between colonoscopy demand and available endoscopic capacity. In this prospective population‐based CRC screening study, we developed an easily implementable decision‐tree model combining f‐Hb with clinical and analytical variables to stratify the risk of CRC and advanced‐stage CRC. The model identified small subgroups at very high and low risk, enabling risk‐based colonoscopy prioritization.

Key strengths include: (I) a large, well‐characterized cohort with independent derivation and validation datasets; (II) the development of a model that promotes equity by not incorporating sex as a predictor, avoiding systematic prioritization of men; (III) identification of a very high‐risk subgroup comprising only 6.7% of FIT‐positive individuals, a proportion that can be feasibly prioritized with existing endoscopy capacity (Figure S2); (IV) substantially lower NNS for CRC and advanced CRC than standard screening approaches [25]; (V) identification of a low‐risk subgroup (13.8%) in whom colonoscopy deferral may be considered individually. TRIPOD recommendations were strictly followed (Supporting Information) [26].

Compared to the overall prevalence of CRC (5.5%) and advanced‐stage disease (1.5%), the algorithm revealed a gradient of tumor risk. The very high‐risk group exhibited a 4–5‐fold higher prevalence of CRC and advanced‐stage disease, whereas the low‐risk group showed a 4–5‐fold lower prevalence. This approach aligns with recommendations supporting risk‐based stratification in CRC screening to enhance program efficiency and may also improve patient satisfaction given the high levels of dissatisfaction associated with colonoscopy waiting times [12, 27].

The Oshima study previously explored a risk‐stratified screening using a two‐sample FIT strategy and clinical risk score to improve sensitivity for advanced colorectal neoplasia, particularly advanced adenomas, in the screening entry phase [28]. In contrast, our study focused exclusively on FIT‐positive individuals and aimed to prioritize colonoscopy according to CRC risk. Because our study was conducted within a single‐sample FIT program, external validation in settings using two‐sample FIT strategies is required.

Our findings reinforce the central role of quantitative f‐Hb concentration as a predictor of CRC risk [13, 14, 15]. Previous models from Italy and Denmark also incorporated f‐Hb but differ substantially from our approach (Table S8) [16, 29]. The Italian model includes sex and classified 23% of participants as high risk, potentially limiting practical prioritization [16]. In contrast, our algorithm identified only 6.7% as very high risk using f‐Hb and age alone. The Danish model combined f‐Hb with multiple blood‐based biomarkers, increasing complexity and cost [29]. By comparison, our model enables identification of the very high‐risk subgroup without blood biomarkers, which are only required for refinement of the low‐risk branch.

The predictive value of f‐Hb and age has also been demonstrated in symptomatic populations [30, 31]. Outside CRC screening settings, established alarm features such as rectal bleeding and iron‐deficiency anemia are associated with NNS values of 5 and 7, respectively, while change in bowel habits or weight loss yield higher NNS values of 20 and 23, respectively [32]. In comparison, our model achieved an overall NNS of 4.6 for CRC and an overall NNS of 13.2 for advanced‐stage CRC, outperforming common alarm symptom‐based colonoscopy indications.

From an implementation perspective, the model should be considered a post‐FIT‐positive triage tool rather than a replacement for FIT‐based screening. FIT‐positive individuals with high f‐Hb and age > 65 years could be prioritized directly for colonoscopy, while routine pre‐endoscopy laboratory tests, particularly ALP and CEA, may further refine risk stratification. Although the algorithm performance was consistent across derivation and validation cohorts, the smaller validation sample resulted in fewer outcome events. Consequently, findings related to advanced‐stage CRC should be considered exploratory and require confirmation in larger external cohorts.

On the low‐risk branch of the algorithm, participants with low f‐Hb and ALP > 105 U/L demonstrated a comparatively low risk of CRC. The NNS among individuals classified as low risk was 14‐fold higher than in the very high‐risk group, indicating greater risk discrimination than previous studies (Table S8). Although ALP emerged as an important CART splitting variable, the biological significance of this observation remains unclear and warrants further investigation. Previous studies have reported decreased ALP levels in patients with CRC, and a low ALP has also been associated with a poorer prognosis in advanced‐stage CRC [33]. Nevertheless, one advanced‐stage CRC occurred within the low‐risk group, highlighting that low‐risk classification should not be interpreted as exclusion of CRC at the individual level.

The proposed risk‐stratification model should not be applied to FIT‐positive individuals receiving anticoagulants, as anticoagulation may increase f‐Hb concentration and lead to misclassification [34]. Importantly, the median f‐Hb value of participants receiving oral anticoagulant therapy in the COLONPREV trial was close to the f‐Hb threshold identified in our model (approximately 100 μg/g). Likewise, individuals classified as very high risk who undergo incomplete colonoscopy or inadequate bowel preparation should undergo repeat colonoscopy as early as possible.

Several limitations should be acknowledged. Although prospectively developed and validated, both cohorts originated from a single center, limiting generalizability. Social determinants of health and other potentially relevant risk factors were not evaluated [35]. Furthermore, the number of outcome events in the validation cohort was limited. Therefore, external multicenter validation is required before implementation across diverse screening settings. Future longitudinal and cost‐effectiveness studies are also needed to assess the clinical impact of risk‐based colonoscopy prioritization. Finally, a universally standardized risk‐stratification model for CRC screening may be difficult to achieve given differences in CRC epidemiology, FIT thresholds, screening strategies, and healthcare resources across countries. Therefore, context‐specific external validation will be essential.

In conclusion, we developed a decision‐tree model for FIT‐positive individuals that identifies small subgroups at very high and low risk of CRC and advanced‐stage CRC. This approach may improve allocation of colonoscopy resources by prioritizing FIT‐positive individuals according to risk. External multicenter validation and cost‐effectiveness analyses are required before widespread implementation.

## Author Contributions

M.C.‐L., C.Q.‐T., A.C.‐C., A.P., and D.M.‐A.: study concept and design. M.C.‐L., C.Q.‐T., C.G.‐M., I.L., P.A., S.C.‐C., S.G.‐M., M.P.‐R., I.P.‐M., and D.M.‐A.: acquisition of the data. C.A.‐C.: histological analysis. M.C.‐L., L.S.‐P., J.M.C.‐S., and D.M.‐A.: statistical analysis and interpretation of data. M.C.‐L.: drafting of manuscript. C.Q.‐T., L.S.‐P., J.M.C.‐S., C.G.‐M., I.L., C.A.‐C., P.A., S.C.‐C., S.G.‐M., M.P.‐R., I.P.‐M., A.C.‐C., A.P., and D.M.‐A.: critical revision of the manuscript. All authors approved the final version of the article. Guarantor of the article: David Marti‐Aguado.

## Funding

This study was funded by Spanish Society of Digestive Endoscopy (SEED) (Grant No. 2021/373). The funding body had no role in study design, data collection, data analysis, the manuscript preparation, or the decision to submit the manuscript for publication.

## Ethics Statement

Approval of the research protocol by an Institutional Reviewer Board: Study protocol was approved by the Clinic University Hospital of Valencia Ethical Committee (Registry number: 2021/373). The protocol conforms to the ethical guidelines of the 1975 Declaration of Helsinki.

## Consent

Written informed consent was obtained from each participant included in the study.

## Conflicts of Interest

The authors declare no conflicts of interest.

## Supporting information


**Table S1:** Prevalence of colorectal cancer according to the delay between a positive fecal immunochemical test (FIT‐positive) and colonoscopy.
**Table S3:** Baseline characteristics and endoscopic findings according to fecal hemoglobin quartiles.
**Table S4:** Performance of the decision‐tree in the very high‐risk group across derivation, validation, and overall cohorts for advanced‐stage colorectal cancer.
**Table S5:** Performance of the decision‐tree model in the high and very high‐risk groups across derivation, validation, and overall cohorts for colorectal cancer.
**Table S7:** Performance of the decision‐tree in the low‐risk group across derivation, validation, and overall cohorts for colorectal cancer.
**Table S8:** Comparison between studies that developed algorithms based on risk stratification for colorectal cancer screening in fecal immunochemical test‐positive individuals.
**Figure S1:** Simplified decision‐tree model defining six colorectal cancer (CRC) risk groups.
**Figure S2:** Surveillance strategies. (A) Current guidelines recommend uniform surveillance for all participants. (B) The proposed risk‐based surveillance allows to prioritize those individuals at higher risk of CRC, optimizing the use of endoscopic resources. CRC, colorectal cancer.

## Data Availability

The data that support the findings of this study are available on request from the corresponding author. The data are not publicly available due to privacy or ethical restrictions.
