# Supplementary material for: Risk Stratification to Optimize Colorectal Cancer Screening: Development and Validation of a Decision‐Tree Model for Colonoscopy Prioritization
Source: Dig Endosc. 2026 Jul 15;38(7):e70224. doi: 10.1111/den.70224 (PMC13373485; doi:10.1111/den.70224)
Supplement: Supplementary file 1 — Table S1: Prevalence of colorectal cancer according to the delay between a positive fecal immunochemical test (FIT‐positive) and colonoscopy. Table S3: Baseline characteristics and endoscopic findings according to fecal hemoglobin quartiles. Table S4: Performance of the decision‐tree in the very high‐risk group across derivation, validation, and overall cohorts for advanced‐stage colorectal cancer. Table S5: Performance of the decision‐tree model in the high and very high‐risk groups across derivation, validation, and overall cohorts for colorectal cancer. Table S7: Performance of the decision‐tree in the low‐risk group across derivation, validation, and overall cohorts for colorectal cancer. Table S8: Comparison between studies that developed algorithms based on risk stratification for colorectal cancer screening in fecal immunochemical test‐positive individuals. Figure S1: Simplified decision‐tree model defining six colorectal cancer (CRC) risk groups. Figure S2: Surveillance strategies. (A) Current guidelines recommend uniform surveillance for all participants. (B) The proposed risk‐based surveillance allows to prioritize those individuals at higher risk of CRC, optimizing the use of endoscopic resources. CRC, colorectal cancer. [file DEN-38-0-s001.docx]

**SUPPLEMENTARY MATERIAL**

1. Supplementary methods: pages 2-3.
2. Supplementary results: page 4
3. Supplementary references: page 5
4. Supplementary tables: pages 6-15.
5. Supplementary figures legend: page 16.
6. Supplementary figures: page 17-19.
7. TRIPOD - Transparent Reporting of Multivariable Prediction Model for Individual: pages 20-23

**SUPPLEMENTARY METHODS:**

Before screening colonoscopy, all participants underwent a standardized research questionnaire and a comprehensive biochemical profile.

The questionnaire recorded smoking status, body mass index (BMI), fecal immunochemical test (FIT) participation round (first or subsequent) and cardiometabolic risk factors. The presence of arterial hypertension, dyslipidemia and diabetes were checked by documented medical history and/or current treatment. Clinical and demographic data included age, sex, cigarette smoking and metabolic comorbidities (diabetes mellitus, dyslipidemia, obesity and hypertension).

Laboratory tests were retrieved from the hospital’s clinical management information system. Serum parameters collected were: glucose (mg/dL), creatinine (mg/dL), hemoglobin (g/dL), serum iron (μg/dL), soluble transferrin receptor (sTfR) (mg/L), cholesterol (mg/dL), triglycerides (mg/dL), high-density lipoprotein cholesterol (HDL-C) (mg/dL), low-density lipoprotein cholesterol (LDL-C) (mg/dL), bilirubin (mg/dL), aspartate aminotransferase (AST) (U/L), alanine aminotransferase (ALT) (U/L), gamma-glutamyl transferase (GGT) (U/L), alkaline phosphatase (ALP) (U/L), leukocytes (x10^3/μL), platelet count (x10^3/μL), international normalized ratio (INR), carcinoembryonic antigen (CEA) (ng/mL). Liver biochemistry variables were included in the blood test profile because of emerging evidence suggesting an association between steatotic liver disease and CRC risk^1.^ Biochemical parameters were measured using automated analyzers routinely employed in the Clinical Biochemistry Laboratory (Cobas platform, Roche Diagnostics, Basel, Switzerland).

**Definition of endoscopic lesions**

Advanced neoplasia was defined as the presence of CRC and/or advanced adenoma (adenoma ≥10 mm and/or with villous component >25%, and/or high-grade dysplasia) and/or advanced serrated lesion (serrated ≥10 mm and/or dysplasia).

**Statistical Analysis**

Categorical variables were expressed as counts and percentages, and continuous variables as medians with interquartile ranges (IQR). Distribution of continuous variables was assessed using the Kolmogorov–Smirnov test. Comparisons between groups were evaluated using the Mann–Whitney U test or the Kruskal–Wallis test for continuous variables, and with the χ² test or Fisher’s exact test for categorical variables.

**SUPPLEMENTARY RESULTS:**

**Prioritization of very high and high-risk groups: derivation cohort**

Expanding prioritization to include both very high- and high-risk groups identified 12.0% (n=140) of participants. In these subjects, the prevalence of CRC and advanced-stage CRC were 18.6% and 7.1%, respectively. Compared to non-prioritized participants, this group showed an OR 5.0 (95%CI 3.0–8.4) for CRC and an OR 13.1 (95%CI 4.7–36.6) for advanced-stage CRC. Prioritizing both very high and high-risk patients entails an NNS of 5.4 to diagnose one case of CRC and an NNS of 14 to diagnose one case of advanced-stage CRC (**Supplementary Table 5**).

**Prioritization of very high and high-risk groups: validation cohort**

Expanding prioritization criteria in the validation cohort identified 14.7% (n=89) of participants. In these subjects, the prevalence of CRC and advanced-stage CRC were 14.6% and 4.5%, respectively. Compared to non-prioritized participants, this group showed an OR 6.6 (95% CI 3.0–14.8) for CRC and an OR 4.8 (95% CI 1.3–18.3) for advanced-stage CRC. Prioritizing both very high and high-risk patients entails an NNS of 6.8 to diagnose one case of CRC and an NNS of 22.3 to diagnose one case of advanced-stage CRC (**Supplementary Table 6**).

**Advanced-stage CRC within the low-risk group**

The only case in the overall study sample with an advanced-stage CRC identified within the low-risk group was a 58-year-old woman with rectosigmoid adenocarcinoma and stage III at diagnosis. Her f-Hb was 22 µg/g, CEA was 30.2 ng/mL, BMI of 21 kg/m^2^ and ALP was 221 U/L. Therefore, this case followed the low f-Hb branch of the decision tree, where ALP >105 U/L led to a low-risk classification. This case illustrates the inherent limitations of hierarchical decision-tree models, in which early branch allocation may outweigh subsequent high-risk features.

**SUPPLEMENTARY REFERENCES**

1. Kimura T, Tamaki N, Wakabayashi SI, et al. Colorectal Cancer Incidence in Steatotic Liver Disease (MASLD, MetALD, and ALD). *Clin Gastroenterol Hepatol*. 2025;23(12):2197-2204.e2. https://doi.org/10.1016/j.cgh.2024.12.018.

**SUPPLEMENTARY TABLES:**

**Supplementary Table 1.** *Prevalence of colorectal cancer according to the delay between a positive fecal immunochemical test (FIT-positive) and colonoscopy.*

| Time between FIT-positive and colonoscopy | Participants (n, %) | CRC (n, %) | Advanced stage  CRC (n, %) *†* |
| --- | --- | --- | --- |
| - ≤ 1 month | 41 (2.3%) | 2 (4.9%) | 0 (0.0%) |
| - 1 – 3 months | 930 (52.5%) | 48 (5.2%) | 16 (1.7%) |
| - 3 – 6 months | 706 (39.8%) | 41 (5.8%) | 7 (1.0%) |
| - > 6 months | 95 (5.4%) | 6 (6.3%) | 2 (2.1%) |

*Note: Values are presented as numbers (%). † Advanced-stage CRC was defined as stage III-IV.*

*Abbreviation: CRC, colorectal cancer.*

**Supplementary Table 2**. Patient characteristics according to the presence or absence of colorectal cancer in the overall cohort.

| Variable | Without CRC  (n = 1,676) | With CRC  (n = 97) | P value |
| --- | --- | --- | --- |
| Female sex | 813 (48.5%) | 43 (44.3%) | 0.42 |
| Age (years) | 61 (56 – 66) | 64 (59 – 67) | 0.001 |
| Tobacco |  |  | 0.03 |
| - Non-smoker | 819 (48.9%) | 55 (56.7%) |  |
| - Ex-smoker | 375 (22.4%) | 26 (26.8%) |  |
| - Current smoker | 482 (28.8%) | 16 (16.5%) |  |
| BMI (kg/m^2^) | 26.8 (24.1 – 30.1) | 27.2 (24.6 – 30.6) | 0.14 |
| Comorbidities |  |  |  |
| - Arterial hypertension | 688 (41.1%) | 43 (44.3%) | 0.52 |
| - Dyslipidemia | 943 (56.3%) | 59 (60.8%) | 0.38 |
| - Diabetes mellitus | 256 (15.3%) | 19 (19.6%) | 0.25 |
| - Obesity | 411 (24.5%) | 29 (29.9%) | 0.23 |
| FIT participation round |  |  | 0.19 |
| - First round | 604 (36.0%) | 46 (47.4%) |  |
| - Subsequent round | 1072 (64.0%) | 51 (52.6%) |  |
| f-Hb (µg/g) | 45.6 (28.6 – 105.0) | 111.8 (53.0 – 200.0) | <0.001 |
| Glucose (mg/dL) | 90 (82 – 101) | 95 (86 – 106) | 0.002 |
| Creatinine (mg/dL) | 0.80 (0.68 – 0.90) | 0.80 (0.68 – 0.96) | 0.85 |
| Hemoglobin (g/dL) | 14.8 (13.9 – 15.7) | 14.7 (13.5 – 15.8) | 0.16 |
| Serum iron (μg/dL) | 91 (72 – 113) | 83 (67 – 111) | 0.14 |
| sTfR (mg/L) | 1.16 (1.01 – 1.38) | 1.20 (1.02 – 1.42) | 0.18 |
| Cholesterol (mg/dL) | 194 (166 – 218) | 190 (151 – 213) | 0.09 |
| Triglycerides (mg/dL) | 114 (85 – 158) | 124 (89 – 166) | 0.34 |
| HDL-C (mg/dL) | 54 (46 – 64) | 49 (42 – 60) | 0.001 |
| LDL-C (mg/dL) | 118 (96 – 140) | 113 (88 – 145) | 0.35 |
| Bilirubin (mg/dL) | 0.64 (0.50 – 0.88) | 0.61 (0.48 – 0.83) | 0.49 |
| AST (U/L) | 24 (21 – 30) | 23 (20 – 30) | 0.49 |
| ALT (U/L) | 21 (17 – 29) | 20 (17 – 28) | 0.27 |
| GGT (U/L) | 26 (19 – 40) | 27 (19 – 38) | 0.95 |
| ALP (U/L) | 78 (66 – 93) | 74 (63 – 89) | 0.13 |
| Leukocytes (x10^3/μL) | 7.23 (6.12 – 8.74) | 6.97 (6.07 – 8.51) | 0.56 |
| Platelet count (x10^3/μL) | 249 (210 – 295) | 252 (213 – 299) | 0.78 |
| INR | 1.00 (1.00 – 1.02) | 1.00 (1.00 – 1.03) | 0.70 |
| CEA (ng/mL) | 2.0 (1.6 – 3.1) | 2.5 (1.7 – 4.0) | <0.001 |

*Note: Values are presented as median (interquartile range) or as number (%).*

*Abbreviations: ALP, alkaline phosphatase; ALT, alanine aminotransferase; AST, aspartate aminotransferase; BMI, body mass index; CEA, carcinoembryonic antigen; CRC, colorectal cancer; f-Hb, fecal hemoglobin; FIT, fecal immunochemical test;* *GGT, gamma-glutamyl transferase; HDL-C, high-density lipoprotein cholesterol; INR, international normalized ratio; LDL-C, low-density lipoprotein cholesterol; sTfR, soluble transferrin receptor.*

**Supplementary Table 3**. Baseline characteristics and endoscopic findings according to fecal hemoglobin quartiles.

| Variable | 20.0 – 29.1 µg/g (n=443) | 29.2 – 47.0 µg/g (n=443) | 47.2 – 110.8 µg/g (n=443) | >110.8 µg/g (n=444) | P value |
| --- | --- | --- | --- | --- | --- |
| - CRC | 7 (1.6%) | 14 (3.2%) | 27 (6.1%) | 49 (11.0%) | <0.001 |
| - Advanced-stage CRC*†* | 2 (0.5%) | 2 (0.5%) | 6 (1.4%) | 15 (3.4%) | <0.001 |

*Note: Values are presented as number (%). † Advanced-stage CRC was defined as stage III-IV.*

*Abbreviation: CRC, colorectal cancer.*

**Supplementary Table 4**. *Performance of the decision-tree in the very high-risk group across derivation, validation and overall cohorts for advanced-stage colorectal cancer.*

| Diagnostic metrics | Derivation cohort | Validation cohort | Overall cohort |
| --- | --- | --- | --- |
| Sensitivity (%) | 43.8 (19.8 – 70.1) | 22.2 (2.8 – 60.0) | 36.0 (18.0 – 57.5) |
| Specificity (%) | 94.7 (93.2 – 95.9) | 91.8 (89.3 – 93.9) | 93.7 (92.5 – 94.8) |
| PPV (%) | 10.3 (4.2 – 20.1) | 3.9 (0.5 – 13.5) | 7.6 (3.5 – 13.9) |
| NPV (%) | 99.2 (98.5 – 99.6) | 98.7 (97.4 – 99.5) | 99.0 (98.4 – 99.5) |
| Correctly classified (%) | 94.0 (92.5 – 95.3) | 90.8 (88.2 – 92.9) | 92.9 (91.6 – 94.1) |
| NNS | 9.7 | 25.5 | 13.2 |
| FP rate (%) | 5.3 | 8.2 | 6.3 |
| FN rate (%) | 56.3 | 77.8 | 64.0 |
| LR+ | 8.3 (4.5 – 15.1) | 2.7 (0.8 – 9.5) | 5.7 (3.3 – 9.9) |
| LR- | 0.6 (0.4 – 0.9) | 0.8 (0.6 – 1.2) | 0.7 (0.5 – 0.9) |
| Odds ratio | 13.9 (5.0 – 38.6) | 3.2 (0.6 – 15.8) | 8.4 (3.6 – 19.4) |

*Note: Values are expressed as percentages (95% confidence interval, CI) unless otherwise indicated. Abbreviations: FN, false negative; FP, false positive; LR+, positive likelihood ratio; LR-, negative likelihood ratio; NNS, number needed to scope; NPV, negative predictive value; PPV, positive predictive value.*

**Supplementary Table 5.** *Performance of the decision-tree model in the high and very high-risk groups across derivation, validation and overall cohorts for colorectal cancer.*

| Diagnostic metrics | Derivation cohort | Validation cohort | Overall cohort |
| --- | --- | --- | --- |
| Sensitivity (%) | 36.6 (25.5 – 48.9) | 50.0 (29.9 – 70.1) | 40.2 (30.4 – 50.7) |
| Specificity (%) | 89.6 (87.6 – 91.3) | 86.9 (83.9 – 89.5) | 88.7 (87.1 – 90.1) |
| PPV (%) | 18.6 (12.5 – 26.0) | 14.6 (8.0 – 23.7) | 17.0 (12.4 – 22.5) |
| NPV (%) | 95.6 (94.2 – 96.8) | 97.5 (95.7 – 98.7) | 96.2 (95.2 – 97.1) |
| Correctly classified (%) | 86.4 (84.3 – 88.3) | 85.3 (82.2 – 88.0) | 86.0 (84.3 – 87.6) |
| NNS | 5.4 | 6.8 | 5.9 |
| FP rate (%) | 10.4 | 13.1 | 11.3 |
| FN rate (%) | 63.4 | 50.0 | 59.8 |
| LR+ | 3.5 (2.5 – 5.0) | 3.8 (2.5 – 5.9) | 3.5 (2.7 – 4.7) |
| LR- | 0.7 (0.6 – 0.8) | 0.6 (0.4 – 0.8) | 0.7 (0.6 – 0.8) |
| Odds ratio | 5.0 (3.0 – 8.4) | 6.6 (3.0 – 14.8) | 5.3 (3.4 – 8.1) |

*Note: Values are expressed as percentages (95% confidence interval, CI) unless otherwise indicated. Abbreviations: FN, false negative; FP, false positive; LR+, positive likelihood ratio; LR-, negative likelihood ratio; NNS, number needed to scope; NPV, negative predictive value; PPV, positive predictive value.*

**Supplementary Table 6**. *Performance of the decision-tree in the high and very high-risk groups across derivation, validation and overall cohorts for advanced-stage colorectal cancer.*

| Diagnostic metrics | Derivation cohort | Validation cohort | Overall cohort |
| --- | --- | --- | --- |
| Sensitivity (%) | 62.5 (35.4 – 84.8) | 44.4 (13.7 – 78.8) | 56.0 (34.9 – 75.6) |
| Specificity (%) | 88.7 (86.7 – 90.5) | 85.8 (82.7 – 88.5) | 87.7 (86.1 – 89.2) |
| PPV (%) | 7.1 (3.5 – 12.7) | 4.5 (1.2 – 11.1) | 6.1 (3.4 – 10.0) |
| NPV (%) | 99.4 (98.7 – 99.8) | 99.0 (97.8 – 99.7) | 99.3 (98.7 – 99.6) |
| Correctly classified (%) | 88.4 (86.4 – 90.1) | 85.2 (82.1 – 87.9) | 87.3 (85.6 – 88.8) |
| NNS | 14.0 | 22.3 | 16.4 |
| FP rate (%) | 11.3 | 14.2 | 12.3 |
| FN rate (%) | 37.5 | 55.6 | 44.0 |
| LR+ | 5.5 (3.7 – 8.4) | 3.1 (1.5 – 6.7) | 4.6 (3.1 – 6.6) |
| LR- | 0.4 (0.2 – 0.8) | 0.7 (0.4 – 1.2) | 0.5 (0.3 – 0.8) |
| Odds ratio | 13.1 (4.7 – 36.6) | 4.8 (1.3 – 18.3) | 9.0 (4.1 – 20.2) |

*Note: Values are expressed as percentages (95% confidence interval, CI) unless otherwise indicated. Abbreviations: FN, false negative; FP, false positive; LR+, positive likelihood ratio; LR-, negative likelihood ratio; NNS, number needed to scope; NPV, negative predictive value; PPV, positive predictive value.*

**Supplementary Table 7.** *Performance of the decision-tree in the low-risk group across derivation, validation and overall cohorts for colorectal cancer.*

| Diagnostic metrics | Derivation cohort | Validation cohort | Overall cohort |
| --- | --- | --- | --- |
| Sensitivity (%) | 1.4 (0.0 – 7.6) | 7.8 (0.9 – 25.1) | 3.1 (0.6 – 8.8) |
| Specificity (%) | 83.8 (81.5 – 86.0) | 88.9 (86.1 – 91.4) | 85.6 (83.6 – 87.3) |
| PPV (%) | 0.6 (0.0 – 3.1) | 3.0 (0.4 – 10.5) | 1.2 (0.2 – 3.6) |
| NPV (%) | 92.9 (91.1 – 94.4) | 96.6 (93.5 – 97.1) | 93.9 (92.5 – 95.0) |
| Correctly classified (%) | 78.8 (76.4 – 81.1) | 85.5 (82.4 – 88.2) | 81.1 (79.2 – 82.9) |
| NNS | 178.0 | 33.0 | 81.3 |
| FP rate (%) | 16.2 | 11.0 | 14.4 |
| FN rate (%) | 98.6 | 92.3 | 96.9 |
| LR+ | 0.1 (0.0 – 0.6) | 0.7 (0.2 – 2.7) | 0.2 (0.2 – 0.7) |
| LR- | 1.2 (1.1 – 1.2) | 1.0 (0.9 – 1.2) | 1.2 (1.1 – 1.2) |
| Odds ratio | 0.1 (0.0 – 0.5) | 0.7 (0.2 – 2.9) | 0.2 (0.1 – 0.6) |

*Note: Values are expressed as percentages (95% confidence interval, CI) unless otherwise indicated.*

*Abbreviations: FN, false negative; FP, false positive; LR+, positive likelihood ratio; LR-, negative likelihood ratio; NNS, number needed to scope; NPV, negative predictive value; PPV, positive predictive value.*

**Supplementary Table 8.** *Comparison between studies that developed algorithms based on risk stratification for colorectal cancer screening in fecal immunochemical test-positive individuals.*

| *Characteristic* | *Petersen, 2024* | *Frazzoni, 2024* | *Present study* |
| --- | --- | --- | --- |
| Country | Denmark | Italy | Spain |
| Study design | Prospective, multicenter | Prospective, multicenter | Prospective,  single-center |
| Study period | 2014 – 2016 | 2004 – 2019 | 2021 – 2024 |
| Sample size | 1977 | 40276 | 1773 |
| CRC prevalence | 12.1% | 5.4% | 5.5% |
| Advanced-stage CRC prevalence | 3.7% | Unknown | 1.5% |
| Age (median) | 65 | 63 | 65 |
| Men (%) | 61% | 58% | 60% |
| FIT participation | Unknown | 59% first round | 36% first round |
| Variables included in the model | f-Hb ≥600 ng Hb/mL, age, CEA, ferritin, CRP, HE4, IL-8, OPG, hepsin, Cyfra21-1 | f-Hb, age, sex, FIT-round | f-Hb, age, ALP, BMI, CEA |
| Validation | Cross-validation | Temporal validation cohort (2016-2019) | Cross-validation and internal validation cohort |
| Prioritized population | 24.7% | 22.9% | 6.7% |
| CRC prevalence in prioritized population | 34.0% | 20.1% | 22% |
| NNS for CRC in the prioritized population | 3.5 | 10.4 | 5.9 |
| Deferrable population | 75.3% | 40.1% | 13.8% |
| CRC prevalence in deferrable population | 5.0% | 2.4% | 1.2% |
| NNS for CRC in deferrable population | 20.1 | 90.9 | 81.3 |
| NNS-CRC fold increase between prioritized *vs*. deferrable groups | 5.7 | 8.7 | 13.8 |
| Strengths | Insights into the role of emerging biomarkers. | Sample size and temporal validation. | Interpretable, simple, easy-to-use model. |
| Limitations | Enriched cohort (oversampling of CRC cases); high cost and analytical complexity. Excessive proportion of high-risk group. | Excessive proportion of high-risk participants. Model prioritizes males over females. | Single center; requires external validation. |

*Abbreviations: CRC, colorectal cancer; FIT, Fecal Immunochemical Test;* *NNS, number needed to scope; NPV, negative predictive value.*

**SUPPLEMENTARY FIGURES LEGENDS**

**Supplementary Figure 1:** Simplified decision-tree model defining six colorectal cancer (CRC) risk groups.

*Terminal nodes show the weighted probability of CRC and corresponding risk categories. ALP, alkaline phosphatase; BMI, body mass index; CEA, carcinoembryonic antigen; CRC, colorectal cancer; f-Hb, fecal hemoglobin.*

**Supplementary Figure 2:** Surveillance strategies. **2A:** Current guidelines recommend uniform surveillance for all participants. **2B:** The proposed risk-based surveillance allows to prioritize those individuals at higher risk of CRC, optimizing the use of endoscopic resources. CRC, colorectal cancer.

**
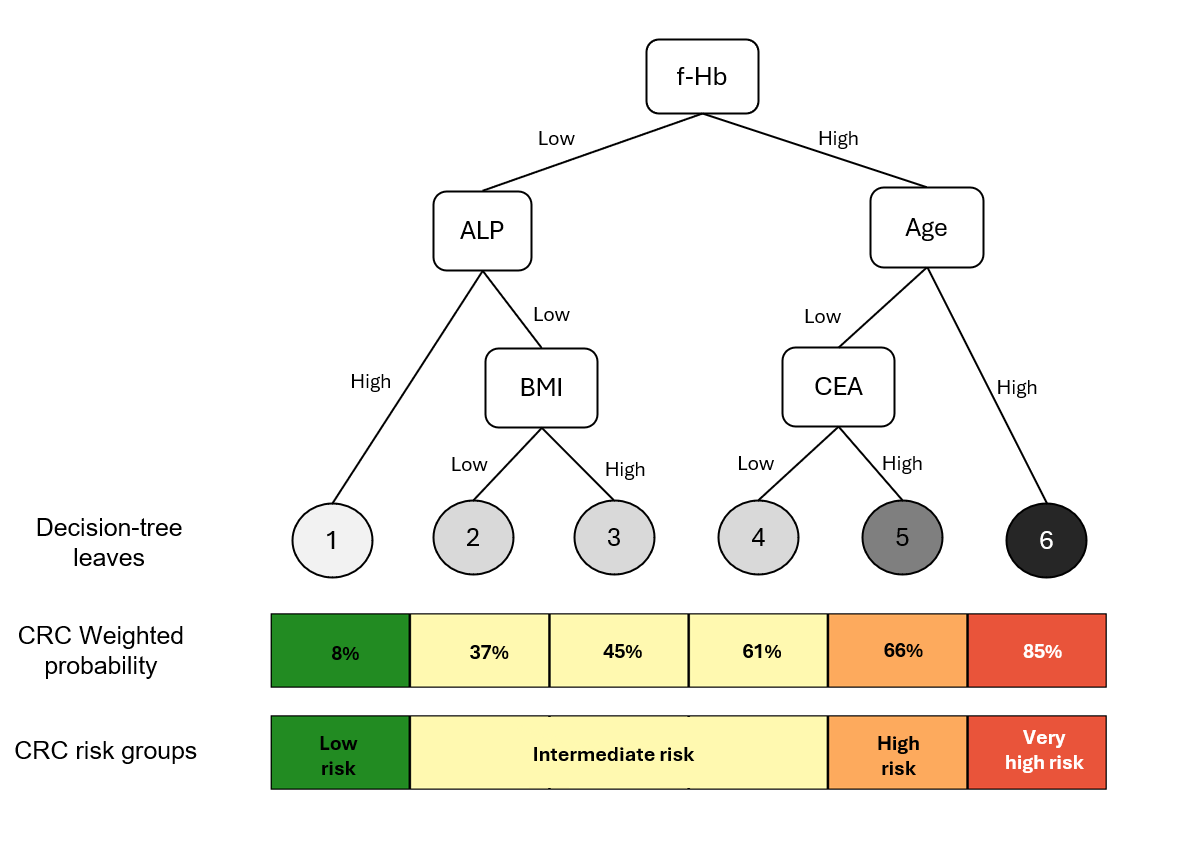
**

**Supplementary Figure 1**

**
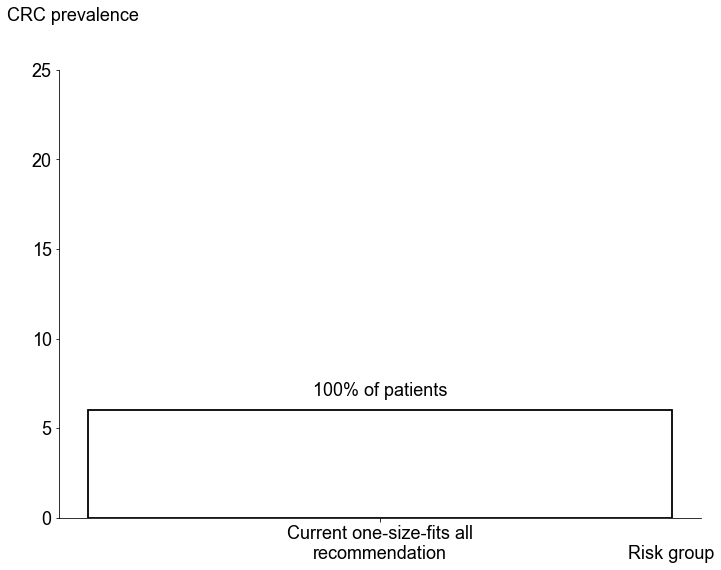
**

**Supplementary Figure 2A**


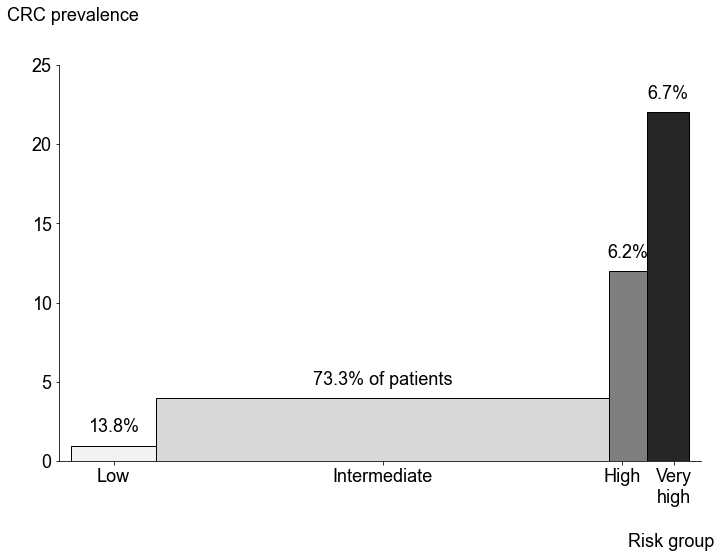


**Supplementary Figure 2B**.

**Transparent Reporting of a Multivariable Prediction Model for Individual Prognosis or Diagnosis (TRIPOD) Checklist for the Predictive Model.**

| **Section/Topic** | **Item** | **Development or Validation** | **Checklist Item** | **Page** |
| --- | --- | --- | --- | --- |
| **Title and Abstract** | | | | |
| Title | 1 | D; V | Identify the study as developing and/or validating a multivariable prediction model, the target population, and the outcome to be predicted. | Main text, page 1 |
| Abstract | 2 | D; V | Provide a summary of objectives, study design, setting, participants, sample size, predictors, outcome, statistical analysis, results, and conclusions. | Main text, page 4,5 |
| **Introduction** | | | | |
| Background and Objectives | 3a | D; V | Explain the medical context (including whether diagnostic or prognostic) and rationale for developing or validating the multivariable prediction model, including references to existing models. | Main text, page 7, 8 |
|  | 3b | D; V | Specify the objectives, including whether the study describes the development or validation of the model or both. | Main text, page 8 |
| **Methods** | | | | |
| Source of Data | 4a | D; V | Describe the study design or source of data (e.g., randomized trial, cohort, registry data), separately for the development and validation data sets, if applicable. | Main text, page 8-10 |
|  | 4b | D; V | Specify the key study dates, including start of accrual; end of accrual; and, if applicable, end of follow-up. | Main text, page 9, 10 |
| Participants | 5a | D; V | Specify key elements of the study setting (e.g., primary care, secondary care, general population) including number and location of centers. | Main text, page 8,9 |
|  | 5b | D; V | Describe eligibility criteria for participants. | Main text, page 8,9 |
|  | 5c | D; V | Give details of treatments received, if relevant. | NA |
| Outcome | 6a | D; V | Clearly define the outcome that is predicted by the prediction model, including how and when assessed. | Main text, page 9-11 |
|  | 6b | D; V | Report any actions to blind assessment of the outcome to be predicted. | NA |
| Predictors | 7a | D; V | Clearly define all predictors used in developing or validating the multivariable prediction model, including how and when they were measured. | Main text, page 9-11; Supplementary Material  page 2 |
|  | 7b | D; V | Report any actions to blind assessment of predictors for the outcome and other predictors. | NA |
| Sample Size | 8 | D; V | Explain how the study size was arrived at. | NA |
| Missing Data | 9 | D; V | Describe how missing data were handled (e.g., complete-case analysis, single imputation, multiple imputation) with details of any imputation method. | Main text, page 11 |
| Statistical Analysis Methods | 10a | D | Describe how predictors were handled in the analyses. | Main text, page 10,11 |
|  | 10b | D | Specify type of model, all model-building procedures (including any predictor selection), and method for internal validation. | Main text, page 10,11 |
|  | 10c | V | For validation, describe how the predictions were calculated. | Main text, page 10,11 |
|  | 10d | D; V | Specify all measures used to assess model performance and, if relevant, to compare multiple models. | Main text, page 10,11 |
| Risk Groups | 11 | D; V | Provide details on how risk groups were created, if done. | Main text, page 10,11; Figure 1 and Supplementary Figure 1 |
| Development vs. Validation | 12 | V | For validation, identify any differences from the development data in setting, eligibility criteria, outcome, and predictors. | Main text, page 10,11 |
| **Results** | | | | |
| Participants | 13a | D; V | Describe the flow of participants through the study, including the number of participants with and without the outcome and, if applicable, a summary of the follow-up time. A diagram may be helpful. | Main text, page 11,12;  Figure 2 |
|  | 13b | D; V | Describe the characteristics of the participants (basic demographics, clinical features, available predictors), including the number of participants with missing data for predictors and outcome. | Main text, page 11,12;  Table 1 |
| Model Development | 14a | D | Specify the number of participants and outcome events in each analysis. | Main text, page 12,13 |
|  | 14b | D | If done, report the unadjusted association between each candidate predictor and outcome. | Supplementary Table 2 |
| Model Specification | 15a | D | Present the full prediction model to allow predictions for individuals (i.e., all regression coefficients, and model intercept or baseline survival at a given time point). | Main Text Page 12,13  Table 3 |
|  | 15b | D | Explain how to use the prediction model. | Figure 3 |
| Model Performance | 16 | D; V | Report performance measures (with confidence intervals) for the prediction model. | Table 2, Supplementary Material  Tables 4-7 |
| Model Updating | 17 | V | If done, report the results from any model updating (i.e., model recalibration) arising from the validation, if applicable. | NA |
| **Discussion** | | | | |
| Limitations | 18 | D; V | Discuss any limitations of the study (such as nonrepresentative sample, few events per predictor, missing data). | Main Text  Page 17 |
| Interpretation | 19a | V | For validation, discuss the results with reference to performance in the development data, and any other validation data. | Main Text  Page 14-17 |
|  | 19b | D; V | Give an overall interpretation of the results, considering objectives, limitations, results from similar studies, and other relevant evidence. | Main Text  Pages 14-17 |
| Implications | 20 | D; V | Discuss the potential clinical use of the model and implications for future research. | Main Text  Pages 14-17 |
| **Other Information** | | | | |
| Supplementary information | 21 | D; V | Provide information about the availability of supplementary resources, such as study protocol, web calculator, and data sets. | Supplementary material,  page 2 |
| Funding | 22 | D; V | Give the source of funding and the role of the funders for the present study. | Main Text, page 18 |

*Note: Items relevant only to the development of a prediction model are denoted by D, items relating solely to a validation of a prediction model are denoted by V, and items relating to both are denoted D; V. We recommend using the TRIPOD Checklist in conjunction with the TRIPOD Explanation and Elaboration document. Abbreviations: NA, not applicable.*
